# Effect of Different Surface Treatments on the Long-Term Repair Bond Strength of Aged Methacrylate-Based Resin Composite Restorations: A Systematic Review and Network Meta-analysis

**DOI:** 10.1155/2022/7708643

**Published:** 2022-09-05

**Authors:** Mahdi Hadilou, Amirmohammad Dolatabadi, Morteza Ghojazadeh, Hossein Hosseinifard, Parnian Alizadeh Oskuee, Fatemeh Pournaghi Azar

**Affiliations:** ^1^Faculty of Dentistry, Tabriz University of Medical Sciences, Tabriz, Iran; ^2^Department of Periodontics, Faculty of Dentistry, Tehran University of Medical Sciences, Tehran, Iran; ^3^Research Center for Evidence-Based Medicine, Tabriz University of Medical Sciences, Tabriz, Iran; ^4^Department of Restorative Dentistry, Faculty of Dentistry, Tabriz University of Medical Sciences, Tabriz, Iran

## Abstract

This systematic review and network meta-analysis is aimed at investigating the effect of common surface treatments on the long-term repair bond strength of aged resin composite restorations and to rank and compare these surface treatments. *In vitro* studies evaluating the methacrylate-based resin composites subjected to rigorous aging protocols before and after being repaired with a new composite were included. A frequentist network meta-analysis was carried out using a random effects model. *P* scores were used to rank the efficacy of the surface treatments. Also, the global and node-split inconsistencies were evaluated. Web of Science, PubMed/Medline, Scopus, and Embase databases were searched until July 07, 2022. Twenty-six studies were included in the meta-analysis. The results showed that the application of silane and a total-etch (shear MD 32.35 MPa, 95% CI: 18.25 to 46.40, *P* score 0.95; tensile MD 33.25 MPa, 95% CI: 25.07 to 41.44; *P* score 0.77) or a self-etch (shear MD 38.87 MPa, 95% CI: 21.60 to 56.14, *P* score 0.99; tensile MD 32.52 MPa, 95% CI: 23.74 to 41.29; *P* score 0.73) adhesion protocol subsequent to the roughening with diamond bur produced the highest (micro)tensile and (micro)shear bond strengths compared to diamond bur alone as the control group. There was no difference between self- and total-etch adhesive protocols. Mechanical surface treatments yielded greater bond strength when used alongside the chemical adhesive agents. Further, it is possible to achieve acceptable repair bond strength using common dental clinic equipment. Therefore, clinicians could consider repairing old resin composites rather than replacing them.

## 1. Introduction

Resin composite restorations must be repaired or replaced, over different time periods following placement due to secondary caries, cracks, marginal discoloration, gaps, cosmetic improvements, shape corrections, and proximal contact closures [1–3]. Based on the extent of the damage and the seriousness of the defect, repair may be a viable option to replacement. Recent advances in adhesive dentistry [4] and equivalent or even superior clinical survival rates of repaired resin composite restorations than replaced ones [5–7] have led to a paradigm shift from full replacement of slightly defective old resin composite restorations towards repairing the defected portion in accordance with minimally invasive dentistry [6, 8].

This approach offers a number of benefits, including preservation of sound tooth structure, prevention of iatrogenic manipulation of pulp tissue [9], time savings, and decreased clinical treatment expenses for individuals [10]. It also is more accepted across patients than replacement [11, 12]. However, due to the gradual deterioration of the structure of resin composite restorations following water absorption in the oral environment [13, 14] and a decrease in chemical surface activity owing to the unavailability of an oxygen-inhibited layer that provides the unreacted residual monomers as a key element in the bonding procedure [15, 16], there are obstacles to attain a satisfactory bond between new and old resin composite restorations.

Patient-related variables (oral hygiene, caries risk, and occlusal forces) and dentist-related ones (experience, technique, and material selection) impact the clinical performance of fresh to old resin composite bond strength [17–19]. The surface treatment protocols performed on old resin composites also play a significant role in determining the durability of the repair bond. *In vitro* research findings had also utilized a variety of surface treatment methods, such as roughening with abrasive papers [20–23], diamond burs [24–26], Al_2_O_3_ particles [3, 22, 27], silica-coated particles [28–30], and lasers [31, 32] as mechanical, and numerous bonding mechanisms, adhesives, and silanization as chemical surface treatments [3, 22, 23, 27], solely or in combination to overcome the bonding obstacles. The main objective of such surface modifications is to produce a strong bond through the micromechanical interlocking between fresh and old resin composites.

The quantity and type of enforced aging also influence the durability and sustainability of repair bond strength of aged resin composites. This process of aging should reflect the fundamental mechanical and chemical properties of the oral environment, such as temperature and pH variations, masticatory forces, and oral habits [33]. Valente et al. [34] presented in 2016 the most recent systematic review and meta-analysis investigating the influence of various surface treatments on the repair bond strength of aged resin composites. Their results were largely derived on relatively short-term static water storage aging tests; thus, they would not have adequately described the long-term repair bond characteristics. To the best of our knowledge, a systematic review and network meta-analysis assessing the influence of common surface treatment approaches on the long-term repair bond strength of properly aged resin composite restorations has still not been conducted. Further, in the lack of head-to-head trials, no research has compared the common surface treatments.

A network meta-analysis offers clinicians with such a rating of surface treatments that may motivate them to repair resin composite restorations with minor defects rather replacing them. In addition, a well-established guideline has still not been developed. This systematic review and network meta-analysis was conducted to offer an explanation for “Which surface treatment strategy improves the long-term repair bond strength of aged methacrylate-based resin composite restorations?”

## 2. Material and Methods

This research is conducted in accordance with the Cochrane Handbook for Intervention Reviews [35] and Preferred Reporting Items for Systematic Reviews and Meta-Analyses (PRISMA) extension for network meta-analyses [36] (Supplementary material 1). The study protocol is registered in the International Prospective Register of Systematic Reviews database (registration code CRD42022308586). A preprint version of this manuscript is available at the medRxiv database [37].

### 2.1. Eligibility Criteria

The studies were included based on the following PICO:

- Population: aged direct or indirect methacrylate-based resin composites (thermocycled at least for 5000 cycles or stored in water at least for 4 weeks). The aging procedure should have been carried out both before surface treatment (primary aging) and after repairing the aged resin composite (secondary aging). The control group's resin composites must have likewise undergone the aging process.
- Intervention: chemical and physical surface treatments including roughening with lasers, diamond burs, Al_2_O_3_, or silica-coated alumina particles and administration of adhesives or silanes, etc. The included papers required a minimum of two comparison groups.
- Comparison: grinding with diamond bur was determined as the control group since it is a common surface treatment used in a clinical setup.
- Outcome: repair bond strengths including tensile, shear, microtensile, and microshear bond strengths.

Studies using nonmethacrylate-based resin composites such as resin nanoceramics, polymer-infiltrated ceramics, and silorane-based resin composites or uncommon aging methods such as UV aging were excluded. Only English language publications were included.

### 2.2. Information Sources and Search

A comprehensive search was performed by one of the authors (MH) up to July 07, 2022, in 4 databases, namely, PubMed/Medline, Embase, Scopus, and Web of Science, using the search strategies outlined in Supplementary material 2. The search strategies consisted of free keywords, Medical Subject Headings (MeSH) terms, and Emtree keywords, combined with the OR and AND Boolean operators. The asterisk (∗) was used to increase the searching accuracy. There was a search of ProQuest Dissertations & Theses for gray literature. Additionally, the references of the included studies were searched for related literature.

### 2.3. Study Selection and Data Extraction

Two researchers (MH and AD) reviewed the papers separately based on eligibility criteria. First on the basis of the titles and abstracts, next, the full-texts. When conflicts arose, a third researcher (PAO) was consulted. The extraction table was created during the pilot stage of the study including 10 articles. The extraction table included the author, year, type of aged and repaired composites, types of primary and secondary aging, total sample for each group, the type of surface treatment (chemical and mechanical), the method of bond strength test, and the related outcomes.

Two researchers (MH and AD) executed the data extraction in duplicate. In the instance of missing information, the study authors were contacted; if no response was obtained, the data were retrieved conservatively. For example, if a spectrum was given as an outcome of each study group, the bottom end of the spectrum was used to establish the group's sample size. If insufficient information was provided, the research would be discarded.

If several doses or levels of a surface treatment were employed in a paper, the only one most often used in the clinical setup was included in the extraction table. In cases where the bond strength of various kinds of resin composites was recorded, the information of all of them was included in the extraction table. If a study assessed the bond strength of a resin composite using a single surface treatment but under multiple aging methods, the strongest aging technique was reported in the extraction table.

### 2.4. Risk of Bias Assessment

The risk of bias assessment was adapted from the Cochrane Collaboration's tool for assessing risk of bias [38] and previously published studies [34, 39]. Two researchers (MH and AD) conducted the risk of bias assessment separately, resolving discrepancies by consulting the third researcher (FPA). The risk of bias graph and summary was created using the robvis visualization instrument [40].

### 2.5. Statistical Analyses

MG and HH conducted a frequentist network meta-analysis employing a random effects model utilizing R software's net meta module (version 3.6.2; R Foundation for Statistical Computing). Every node indicated an intervention from the articles included in the analysis. The lines linking the nodes reflected present head-to-head comparisons, and their thickness was proportional to the number of studies that examined these comparisons. The surface treatments were ranked according to their respective *P* scores. Higher *P* scores demonstrate a higher likelihood that the intervention is superior to other comparisons [41]. The network-level inconsistency (global inconsistency) was presented by *I*^2^ [42]. In addition, node-split inconsistency was examined by comparing the direct and indirect evaluations. If there was a significant discrepancy (*P* < 0.05) in the direct and indirect evaluations, the direct evaluation was reported as the pooled estimate in the league tables. To identify any publication bias or small study effect, comparison-adjusted funnel plots were created for all possible comparisons with diamond bur serving as the control group.

In the case of reporting the bond strength of multiple types of methacrylate-based composites with the identical intervention, the mean ± standard deviation of these groups was merged into one group. The adhesives were classified as either self-etch or total-etch. In the case of using multiple mechanical surface treatments on a single group of resin composites, all treatments were reported in the extraction table. However, only the dominant physical surface treatment depicted the group over the abrasive paper in the network meta-analysis. Further, the use of phosphoric acid was not considered a surface treatment since it is primarily employed for surface cleaning. Despite its application having a positive impact on enamel and dentin adhesion, it does not influence the shear bond strength of the repaired resin composite, since it is too weak to create sufficient surface irregularities [43].

## 3. Results

**Figure 1:**
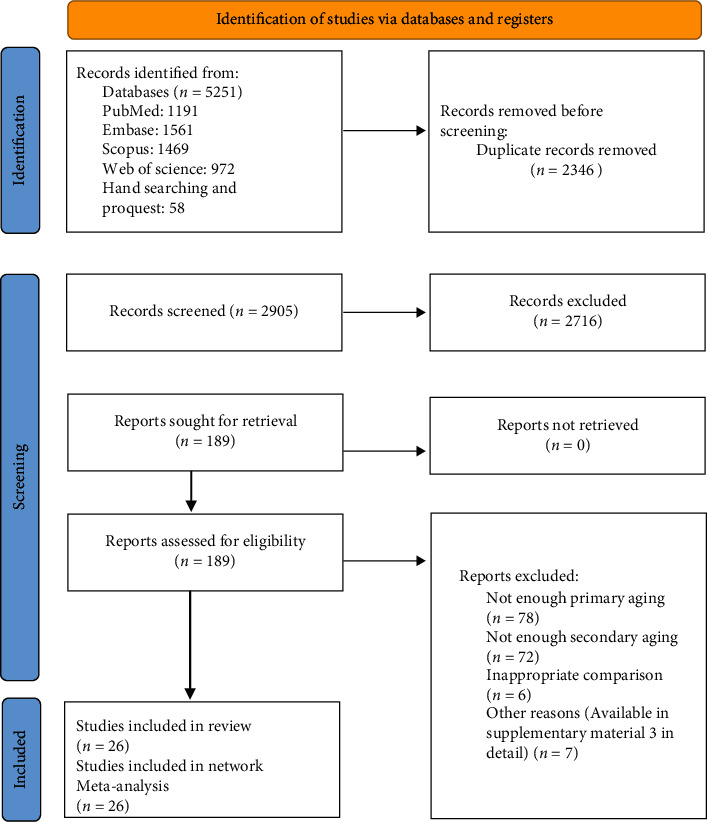
PRISMA flowchart.

The search yielded 5251 papers, of which 2346 were duplicates. After screening based on titles and abstracts, 189 papers were chosen for full-text examination, of which 26 fulfilled the inclusion criteria and were included in the network meta-analysis. The excluded articles and their justifications are available as Supplementary material 3. The PRISMA flowchart is shown in Figure 1.

### 3.1. Characteristics of Included Studies

All twenty-six included studies had *in vitro* lab settings. The publishing dates of the included papers spanned from 2007 to 202*2*. The sample sizes varied between 5 [44] and 66 [45] in each study. The aging methods included liquid storage (water [3, 9, 27, 46–48] and saliva [49]), thermocycling [20, 22–25, 29, 31, 32, 43, 50, 51], or a combination of the two [21, 26, 28, 30, 44, 45, 52, 53]. Ten studies [22–25, 30, 44, 45, 48, 49, 53] provided microtensile bond strength, four [3, 9, 46, 50] indicated microshear bond strength, eleven [20, 21, 26–29, 31, 32, 43, 51, 52] gave shear bond strength, and only one [47] evaluated tensile bond strength (Table 1).

### 3.2. Risk of Bias Assessment

**Figure 2:**
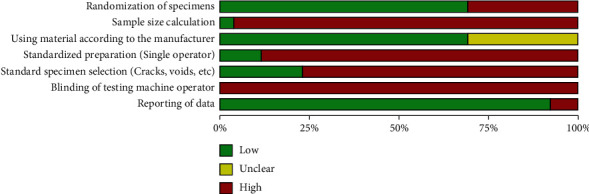
Risk of bias summary.

All of the studies had different levels of bias (Supplementary material 4). The absence of blinding the testing machine operator was the most biased field of the included research. In addition, sample size calculation seemed to be mostly arbitrary across the studies. Furthermore, standard preparation of specimens (by single operator) and standard specimen selection (the procedure of identifying and excluding defective specimens) domains posed a significant risk of bias (Figure 2).

### 3.3. Network Meta-analysis Results

**Figure 3:**
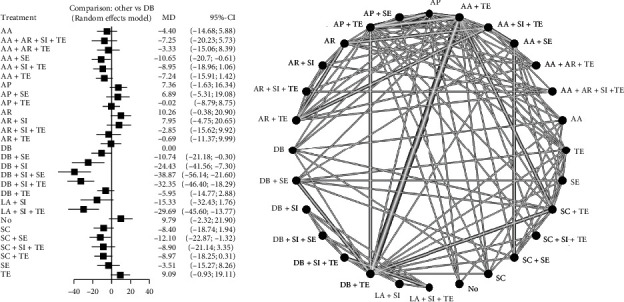
Network map of surface treatments introduced into the network meta-analysis for (micro)shear bond strength by random effects model. AP: abrasive paper; No: no surface treatment; AA: air abrasion with Al_2_O_3_ particles; SI: silane; TE: total-etch adhesive; SE: self-etch adhesive; AR: argon plasma; DB: diamond bur; SC: air abrasion with silica-coated Al_2_O_3_ particles; FRC: flowable resin composite; LA: laser.

**Figure 4:**
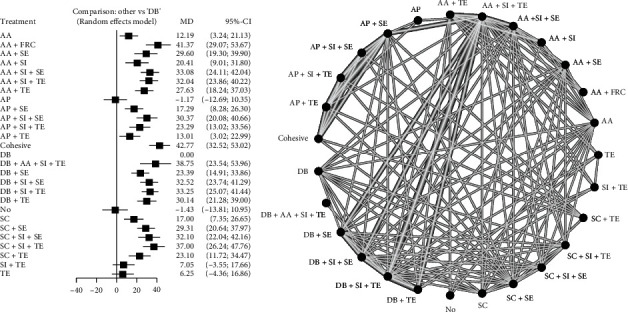
Network map of surface treatments introduced into the network meta-analysis for (micro)tensile bond strength by random effects model. AP: abrasive paper; No: no surface treatment; AA: air abrasion with Al_2_O_3_ particles; SI: silane; TE: total-etch adhesive; SE: self-etch adhesive; AR: argon plasma; DB: diamond bur; SC: air abrasion with silica-coated Al_2_O_3_ particles; FRC: flowable resin composite; LA: laser.

Twenty-six studies were included in the network meta-analysis. The global network heterogeneity was substantial for both (micro)tensile (*I*^2^ = 82.4%) and (micro)shear networks (*I*^2^ = 94.7%). The following surface treatments were used alone or in combination: (1) AP—abrasive paper, (2) No—no surface treatment, (3) AA—air abrasion with Al_2_O_3_ particles, (4) SI—silane, (5) TE—total-etch adhesive, (6) SE—self-etch adhesive, (7) AR—argon plasma, (8) DB—diamond bur (with coarse particles for surface treatment purpose), (9) SC—air abrasion with silica-coated Al_2_O_3_ particles, (10) FRC—flowable resin composite, and (11) LA—laser. Totally, 28 surface treatments were included in the (micro)shear network (Figure 3) and 27 in the (micro)tensile network (Figure 4). Supplementary material 5 contains league tables of mixed-effects pooled estimates.

**Figure 5:**
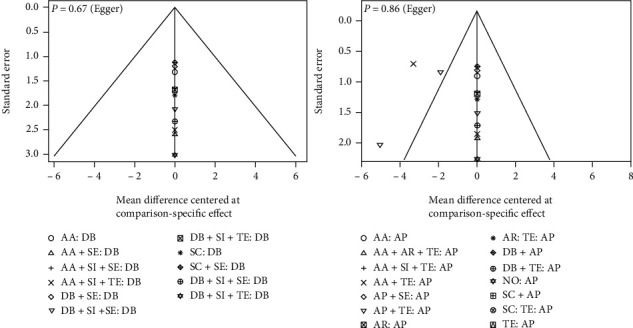
Funnel plots of publication bias tests.

The forest plots are shown in Figures 3 and 4, and Table 2 contains the associated *P* scores. Most head-to-head comparisons were based on a single study. The surface treatment with the greatest chance of producing higher (micro)shear bond strength was DB+SI+SE (*P* score = 0.99), preceded by DB+SI+TE (*P* score = 0.95) and LA+SI+TE (*P* score = 0.92). AA+FRC (*P* score = 0.93) had the greatest likelihood of being the optimal surface treatment for increasing (micro)tensile bond strength, trailed by DB+AA+SI+TE (*P* score = 0.87) and SC+SI+TE (*P* score = 0.86), and ultimately, the two interventions with the greatest likelihood of yielding higher (micro)shear and (micro)tensile bond strengths were DB+SI+TE (shear MD: 32.35 MPa (mega Pascal), 95% CI: 18.25 to 46.40, *P* score = 0.95; tensile MD: 33.25 MPa, 95% CI: 25.07 to 41.44; *P* score = 0.77) and DB+SI+SE (shear MD: 38.87 MPa, 95% CI: 21.60 to 56.14, *P* score = 0.99; tensile MD: 32.52 MPa, 95% CI: 23.74 to 41.29; *P* score = 0.73). Per the Egger's test, there was no publication bias in the included studies of (micro)tensile (*P* = 0.67) and (micro)shear (*P* = 0.86) networks (Figure 5). Evaluation of node-split inconsistency (Supplementary material 6) revealed that 0 out of 94 indirect network estimates in the (micro)shear network and 9 of 106 indirect network estimates in the (micro)tensile network differed from their related direct estimates which reflects very low level of inconsistency of the analyses.

## 4. Discussion

The annual failure rate for resin composite restorations ranges from 1 to 4% [54–56]. Recent advances in adhesive dentistry have made it possible to repair partially defective old resin composite restorations as opposed to their complete replacement, which is fraught with complications [9]. Consequently, this systematic review and network meta-analysis aimed at investigating the effect of common surface treatments on the long-term repair bond strength of aged resin composite restorations and at ranking and comparing these surface treatments.

Valente et al. [34] conducted the most recent systematic review and meta-analysis investigating the effect of different surface treatments on the repair bond strength of aged resin composite restorations. Most of their findings were derived from short-term (24 hours to 2 weeks) static water storage aging, and the surface treatments that demonstrated relative bond strength values to cohesive strength had not been subjected to rigorous aging protocols. Thus, their findings could not accurately represent the long-term repair bond properties, suggesting that future research should employ more stringent aging protocols.

Presently, most published studies examine resin composites' immediate or short-term bond strength and apply mild aging protocols, such as the protocol recommended by ISO/TS 11405:2015 [57, 58]. In contrast, only rigorous aging methods can accurately imitate the oral cavity's inherent conditions, such as exposure to chemical agents of food, saliva, occlusal forces, and changes in temperature and pH [59]. In addition, most studies do not perform secondary aging (after repairing the old composite), even though the bond's clinical relevance and long-term durability rely heavily on the bonded interface, which should also be evaluated using rigorous aging methods [33]. Notably, the 500 cycles of thermocycling recommended by the ISO TR 11450 standard [57, 58] are inadequate to simulate the long-term changes in bonding interface and composite structure degradation [33]. In a recently published study comparing several resin composite aging protocols, Szczesio-Wlodarczyk et al. [60] found that ISO/TR 11450 recommendations had no significant effect on the surface properties or inner structure of resin composites. They observed that 7500 cycles of thermocycling were one of the most effective aging protocols for simulating the oral environment on the strength properties of resin composites. Furthermore, Ghavami-Lahiji et al. [61] demonstrated that changes in the mechanical properties of resin composites began to occur after 4000 thermocycling cycles. As stated previously, researchers have reached a consensus regarding the inadequacy of the ISO/TR 11450-recommended aging protocol. Nonetheless, there is a variety of viewpoints regarding the optimal amount of aging, ranging from 4,000 to 100,000 cycles of thermocycling [62] to three months of storage in water [63]. Based on the evidence presented above and a similar systematic review [64] that led to a sufficient number of studies to perform network meta-analyses, the aging limitation in the current study was determined to be at least one month of storage in water or 5000 cycles of thermocycling.

The diamond bur+silane+self-etch or total-etch adhesive produced the highest (micro)tensile and (micro)shear bond strengths compared to the diamond bur alone as the control. However, compared to cohesive strength, they generated 10.25 MPa (95% CI: 1.18 to 19.33) and 9.25 MPa (95% CI: 1.20 to 17.84) lower (micro)tensile bond strength, respectively, indicating that adhesion protocols to the aged resin composites have room for improvement (Table 2 and Supplementary material 5). Over 80% of dentists are inclined to use the same surface treatment for composite repair as they do for restoration replacement, a roughening with a diamond bur followed by acid etching and adhesives [65]. Therefore, the diamond bur+silane+self-etch or total-etch adhesion protocols are the most practical due to their accessibility, convenience, lower cost, and absence of additional dental equipment.

The application of silane and total-etch adhesion protocols after air abrasion with silica-coated alumina particles (SC+SI+TE) was one of the three most effective techniques for increasing the (micro)tensile bond strength (Table 2). In addition to roughening the surface of old resin composites, silica-coated alumina particles also leave behind a silica-rich layer that promotes bonding via chemical coupling with the subsequent silane [34]. Silanization also improves surface wettability; however, its effect on chemical coupling improvement is dependent on silica's presence on the old composite surface, which can be supplied by silica-coated alumina particles or the structure of resin composites. The latter is improbable, because the deterioration of resin composite structure caused by aging breaks the bond between the filler and polymer, resulting in the erosion of surface glass particles [14].

Laser surface roughening followed by the silanization and total-etch adhesion protocol (LA+SI+TE) was the second-best technique in the (micro)shear network. Recent *in vitro* studies have introduced this technique. None of the included articles examined the laser's effectiveness as a (micro)tensile bond strength measurement tool. Hence, it is suggested that additional research investigate the impact of lasers as surface treatments on the repair bond strength of aged resin composites.

There were no differences between self-etch and total-etch adhesion protocols. In addition, using a chemical substance such as silane or adhesive significantly enhanced the (micro)tensile bond strength. Eight comparisons revealed its effectiveness, while four revealed no difference (Supplementary material 5). This is consistent with Valente et al. [34], who also emphasized the importance of applying chemical surface treatments following mechanical roughening.

According to the provided league tables (Supplementary material 5), the comparisons between groups with silanization and groups with the same surface treatment as the first group but without using silane were inconclusive. Ten of the eighteen comparisons in the (micro)shear and (micro)tensile league tables showed no difference, while eight favored the silane-treated groups. A silane coupling agent is applied to improve the surface wettability of the composite substrate surface. Additionally, it forms covalent bonds with the exposed filler particles on the surface of the old resin composite and copolymerizes with the methacrylate groups of the repair material, leading to bond improvement [25]. Mendes et al. [39] found no difference in the aged resin composite subgroup when only silane or silane+adhesive groups were used as surface treatments in a meta-analysis specifically designed to investigate the effectiveness of silane agents on resin composite repair bond strength. Nonetheless, silane in conjunction with adhesive agents appears to have minimal impact on repair bond strength of aged resin composites.

According to the risk of bias summary (Figure 2), calculating sample size appeared more arbitrary than via power analyses, which may have compromised the external validity of the individual studies' results. In addition, standard sample preparation by a single operator and selection of standard specimens (evaluation of the prepared samples for the presence of microcracks, voids, and deformities) by direct observation or using microscopes were not mentioned in more than 80% of the included studies. In neither of the experiments was the operator of the evaluating machine blinded. Unfortunately, poor adherence to the aforementioned sources of bias is prevalent in laboratory experiments, which significantly impacts the pooled results and should be properly considered in future research. For clinical application, these results should ultimately be interpreted with caution.

## 5. Strengths and Limitations

The current study had several advantages as follows: providing a network meta-analysis that ranked the widely used clinical surface treatments in terms of being best to yield stronger repair-bond-strength of aged resin composite restorations, comparing these surface treatments despite the lack of direct comparisons, determining rigorous aging protocols as inclusion criteria both before and after repairing resin composites better to simulate the oral environment on a long-term scale, and attempting to simulate the oral environment, while seeking to cover more databases compared to the meta-analysis in the literature [34].

However, the study also had certain limitations. Due to the limited number of included studies, most network comparisons were derived from single trials. Although the primary difference between micro- and macro-bond strength depends on the size of the bonded area, pooling the results of micro- and macro-bond strengths due to the small number of included studies may have affected the results.

## 6. Conclusions

In this study, two network meta-analyses identified diamond bur+silane+total-etch or self-etch adhesion protocol as the most efficient techniques for improving (micro)tensile and (micro)shear bond strength. In addition, there was no discernible difference between self-etch and total-etch adhesion protocols. Also, mechanical surface treatments should be followed by applying chemical surface treatments for increased repair bond strength.

## Figures and Tables

**Table 1:** Characteristics of the included studies.

Author (year)	Aged composite	Repair composite	Primary/secondary aging type	Group size	Surface treatment		Test	Bond strength (MPa)
					Mechanical	Chemical		
Ugurlu et al. (2022) [48]	ReliaFIL Bulk ReliaFIL LC	ReliaFIL LC	2 years in water (37°C)/6 months in water (37°C)	40	G1: AA+PhA	SI+AD (ScotchBond Universal) (TE)	*μ*TBS	Bulk: 46.07 ± 5.52 LC: 45.61 ± 4.80
					G2: AA+PhA	SI+AD (All-Bond Universal) (TE)		Bulk: 41.72 ± 5.30 LC: 41.66 ± 5.29
					G3: AA+PhA	AD (ScotchBond Universal) (TE)		Bulk: 40.37 ± 5.16 LC: 40.30 ± 5.09
					G4: AA+PhA	AD (All-Bond Universal) (TE)		Bulk: 38.14 ± 4.75 LC: 37.66 ± 5.19
					G5: DB+PhA	SI+AD (ScotchBond Universal) (TE)		Bulk: 44.50 ± 5.51 LC: 45.24 ± 5.23
					G6: DB+PhA	SI+AD (All-Bond Universal) (TE)		Bulk: 40.58 ± 5.43 LC: 40.91 ± 5.73
					G7: DB+PhA	AD (ScotchBond Universal) (TE)		Bulk: 40.22 ± 5.22 LC: 40.06 ± 5.01
					G8: DB+PhA	AD (All-Bond Universal) (TE)		Bulk: 36.93 ± 5.09 LC: 36.66 ± 5.26
					G9: PhA	SI+AD (ScotchBond Universal) (TE)		Bulk: 17.06 ± 5.04 LC: 18.06 ± 5.26
					G10: PhA	SI+AD (All-Bond Universal) (TE)		Bulk: 16.88 ± 4.44 LC: 17.28 ± 4.99
					G11: PhA	AD (ScotchBond Universal) (TE)		Bulk: 15.86 ± 5.84 LC: 16.91 ± 6.37
					G12: PhA	AD (All-Bond Universal) (TE)		Bulk: 16.67 ± 4.83 LC: 16.68 ± 4.84

Chuenweravanich et al. (2022) [53]	Filtek Z350XT	Filtek Z350XT	1 month in water (37°C)/thermal cycling (10000 cycles, 5–55°C, dwell time: 30 s, transfer time: 4 s)	5	G1: AP	AD (Single Bond Universal) (SE)	*μ*TBS	24.22 ± 3.55
					G2: AP+DB	AD (Single Bond Universal) (SE)		32.26 ± 4.02
					G3: AP+DB+PhA	AD (Single Bond Universal) (TE)		38.46 ± 2.60
					G4: AP+DB	SI+AD (Single Bond Universal) (SE)		46.78 ± 4.99
					G5: AP+DB+PhA	SI+AD (Single Bond Universal) (TE)		48.36 ± 2.44
					G6: AP+AA+PhA	SI+AD (Single Bond Universal) (TE)		38.48 ± 2.78

Burrer et al. (2021) [22]	Ceram.x Spectra ST (HV)	Ceram.x Spectra ST (HV)	Thermal cycling (5000 cycles, 5–55°C, dwell time: 20 s, transfer time: 10 s)/same	8	G1: AP	AD (OptiBond FL) (TE)	*μ*TBS	19.1 ± 13.0
					G2: AP+AA	SI+AD (OptiBond FL) (TE)		37.8 ± 9.5

Karadas (2021) [23]	Charisma Smart	Charisma Smart	Thermal cycling (5000 cycles, 5–55°C, dwell time: 30 s)/same (30000 cycles)	60	G1: AP	AD (All-Bond Universal) (SE)	*μ*TBS	22.99 ± 6.30
					G2: AP	AD (Scotchbond Universal) (SE)		29.79 ± 8.01
					G3: AP	AD (Tokuyama Universal Bond) (SE)		28.82 ± 10.27
					G4: AP	No treatment		6.95 ± 2.85

Negreiros et al. (2021) [3]	Filtek Supreme Ultra	Filtek Supreme Ultra Charisma	4 months in water (37°C)/1 year in water (37°C)	6	G1: AP	No treatment	*μ*SBS	Filtek: 3.6 ± 1.0 Charisma: 1.4 ± 0.3
					G2: AP+AA	SI+AD (Adper Scotchbond MP) (TE)		Filtek: 20.3 ± 2.9 Charisma: 17.7 ± 1.5
					G3: AP	AD (Adper Scotchbond MP) (TE)		Filtek: 20.7 ± 4.2 Charisma: 17.8 ± 1.3
					G4: AP+AR	AD (Adper Scotchbond MP) (TE)		Filtek: 12.8 ± 2.4 Charisma: 14.4 ± 1.4
					G5: AP+AA	AD (Adper Scotchbond MP) (TE)		Filtek: 20.8 ± 3.5 Charisma: 17.9 ± 1.6
					G6: AP+AA+AR	AD (Adper Scotchbond MP) (TE)		Filtek: 15.4 ± 5.6 Charisma: 17.3 ± 1.6
					G7: AP+AR	No treatment		Filtek: 3.2 ± 0.6 Charisma: 4.2 ± 1.2

Willers et al. (2021) [27]	Charisma	Charisma	4 years in water (37°C)/1 year in water (37°C)	18	G1: AP+AA	SI+AD (Adper Scotchbond MP) (TE)	SBS	18.7 ± 3.6
					G2: AP+AA	AD (Gluma Bond Universal) (SE)		14.3 ± 4.6
					G3: AP+AA	AD (Adhese Universal) (SE)		18.6 ± 2.9
					G4: AP+AA	AD (Scotchbond Universal) (SE)		19.8 ± 3.9

Michelotti et al. (2020) [24]	Filtek Supreme XTE	Filtek Supreme XTE	Thermal cycling (5000 cycles, 5–55°C, dwell time: 20 s; transfer time: 10 s; duration of each cycle: 50 s)/same	6	G1: AP+DB	AD (Scotchbond Universal) (SE)	*μ*TBS	27.13 ± 1.24
					G2: AP+DB	SI+AD (Scotchbond Universal) (SE)		31.62 ± 4.36
					G3: AP+DB	SI+AD (OptiBond FL) (TE)		35.43 ± 3.24
					G4: AP+DB	No treatment		2.94 ± 2.78
					G5: AP+AA	AD (Scotchbond Universal) (SE)		32.52 ± 5.74
					G6: AP+AA	SI+AD (Scotchbond Universal) (SE)		37.04 ± 3.03
					G7: AP+AA	SI+AD (OptiBond FL) (TE)		39.88 ± 5.53
					G8: AP+AA	No treatment		12.37 ± 1.73
					G9: AP+SC	AD (Scotchbond Universal) (SE)		33.56 ± 0.50
					G10: AP+SC	SI+AD (Scotchbond Universal) (SE)		35.01 ± 5.09
					G11: AP+SC	SI+AD (OptiBond FL) (TE)		39.97 ± 6.98
					G12: AP+SC	No treatment		19.90 ± 3.58

Moura et al. (2020) [28]	Filtek Z350	Filtek Z350	6 months in water/thermal cycling (10000 cycles; 5–55°C; dwell time: 30 s)	12	G1: AP+SC	AD (Scotchbond Universal) (SE)	SBS	20.92 ± 7.29
					G2: AP+AA	AD (Scotchbond Universal) (SE)		18.18 ± 5.6

Dieckmann et al. (2020) [25]	Filtek Supreme XTE	Filtek Supreme XTE	Thermal cycling (5000 cycles, 5–55°C; dwell time: 20 s; transfer time: 10 s)/same	6	G1: AP	AD (OptiBond FL) (TE)	*μ*TBS	0.00 ± 0.00
					G2: AP+DB	SI+AD (OptiBond FL) (TE)		22.98 ± 5.89
					G3: AP+DB+AA	SI+AD (OptiBond FL) (TE)		28.43 ± 9.86

Kanzow et al. (2019) [51]	Filtek Supreme XTE	Filtek Supreme XTE	Thermal cycling (10000 cycles; 5–55°C; dwell time: 20 s; transfer time: 10 s)/same	16	G1: No treatment	AD (Scotchbond Universal) (SE)	SBS	18.04 ± 6.25
					G2: AP+DB	AD (Scotchbond Universal) (SE)		27.07 ± 4.04
					G3: AP+AA	AD (Scotchbond Universal) (SE)		27.76 ± 4.07
					G4: AP+SC	AD (Scotchbond Universal) (SE)		26.09 ± 3.30
					G5: No treatment	AD (Adper Scotchbond MP) (TE)		2.17 ± 1.87
					G6: AP+DB	AD (Adper Scotchbond MP) (TE)		14.60 ± 5.05
					G7: AP+AA	AD (Adper Scotchbond MP) (TE)		25.01 ± 4.59
					G8: AP+SC	AD (Adper Scotchbond MP) (TE)		25.64 ± 3.91

Ayres et al. (2019) [46]	Charisma	Charisma	6 months in water (37°C)/1 year in water (37°C)	10	G1: AP+AA	SI+AD (Adper Scotchbond MP) (TE)	*μ*SBS	23.0 ± 3.2
					G2: AP+AA+AR	SI+AD (Adper Scotchbond MP) (TE)		20.3 ± 4.3
					G3: AP+AR	SI+AD (Adper Scotchbond MP) (TE)		15.9 ± 2.1
					G4: AP+AR	SI		5.1 ± 0.4
					G5: AP+AR	AD (Adper Scotchbond MP) (TE)		13.8 ± 3.4
					G6: AP+AR	No treatment		1.8 ± 0.6

Demirel and Gur (2019) [50]	Clearfil Majesty Esthetic	Clearfil Majesty Esthetic	Thermal cycling (10000 cycles; 5–55°C; dwell time: 30 s; transfer time: 10 s)/same	11	G1: AP+DB+PhA	SI+AD (Clearfil S3 Bond Plus) (TE)	*μ*SBS	68.85 ± 4.89
					G2: AP+DB+PhA	AD (Single Bond Universal) (TE)		45.90 ± 6.40
					G3: AP+DB+PhA	AD (Clearfil Universal) (TE)		48.91 ± 7.90
					G4: AP+DB+PhA	AD (Clearfil S3 Bond Plus) (TE)		32.36 ± 5.64

Flury et al. (2019) [9]	Filtek Z250	Filtek Z250	3 months in water (37°C)/1 year in water (37°C)	15	G1: AA	SI+AD (OptiBond FL) (TE)	*μ*SBS	13.41 ± 3.25
					G2: AA	AD (Scotchbond Universal) (SE)		14.17 ± 2.87

Oglakci and Arhun (2019) [43]	Tetric EvoCeram Bulk Fill	Tetric EvoCeram Bulk Fill Tetric EvoCeram Nanohybrid	Thermal cycling (5000 cycles, 5–55°C; dwell time: 20 s; transfer time: 10 s)/same	15	G1: AP+DB	AD (Tetric N-Bond Universal) (SE)	SBS	BF: 24.69 ± 4.82 NH: 20.69 ± 7.17
					G2: AP+DB+PhA	AD (Tetric N-Bond Universal) (TE)		BF: 25.86 ± 5.74 NH: 20.41 ± 3.70
					G3: AP+DB	AD (Clearfil SE) (SE)		BF: 27.05 ± 4.93 NH: 22.08 ± 6.37
					G4: AP+DB+PhA	AD (Adper Single Bond 2) (TE)		BF: 24.49 ± 6.95 NH: 18.74 ± 6.40

Kouros et al. (2018) [26]	Filtek Ultimate	Filtek Ultimate	6 months in artificial saliva/thermal cycling (5000 cycles; 5–550°C; dwell time: 30 s)	10	G1: DB+PhA	AD (Adper Single Bond 2) (TE)	SBS	52.72 ± 10.9
					G2: AA+PhA	AD (Adper Single Bond 2) (TE)		55.56 ± 14.76

Peterson et al. (2017) [20]	Venus Diamond	Constic Fusio Liquid Dentin Vertise Flow Venus Diamond	Thermal cycling (5000 cycles; 5–55°C)/same	16	G1: AP+DB	AD (OptiBond FL) (TE)	SBS	(Constic): 12.7 ± 2.7 (Fusio): 19.9 ± 6.4 (Vertise): 12.0 ± 3.0 (Venus): 16.4 ± 7.4
					G2: DB	No treatment		(Constic): 10.9 ± 3.9 (Fusio): 14.7 ± 6.2 (Vertise): 6.5 ± 6.2 (Venus): 5.9 ± 3.3
					G3: AP+AA	AD (OptiBond FL) (TE)		(Constic): 11.7 ± 3.0 (Fusio): 15.1 ± 2.7 (Vertise): 14.2 ± 3.3 (Venus): 17.3 ± 5.1
					G4: AP+AA	No treatment		(Constic): 10.4 ± 2.1 (Fusio): 18.8 ± 5.2 (Vertise): 10.0 ± 3.3 (Venus): 16.6 ± 4.5
					G5: AP+SC	AD (OptiBond FL) (TE)		(Constic): 10.3 ± 3.8 (Fusio): 19.8 ± 5.3 (Vertise): 14.4 ± 4.1 (Venus): 21.3 ± 3.1
					G6: AP+SC	No treatment		(Constic): 13.5 ± 3.6 (Fusio): 21.7 ± 7.4 (Vertise): 12.2 ± 3.9 (Venus): 24.4 ± 2.8
					G7: AP	AD (OptiBond FL) (TE)		(Constic): 10.0 ± 4.3 (Fusio): 15.9 ± 5.5 (Vertise): 9.1 ± 2.4 (Venus): 11.0 ± 5.7
					G8: AP	No treatment		(Constic): 5.6 ± 3.4 (Fusio): 6.4 ± 4.1 (Vertise): 6.5 ± 4.0 (Venus): 0.3 ± 0.6

Souza et al. (2017) [49]	Esthet-X	Esthet-X	1 year in artificial saliva/same	16	G1: no treatment	No treatment	*μ*TBS	11.18 ± 4.86
					G2: AA	No treatment		30.48 ± 13.86
					G3: AA	AD (Adper Scotchbond MP) (TE)		34.96 ± 20.74
					G4: AA	SI		30.67 ± 12.81

Kiomarsi et al. (2017) (a) [31]	Filtek Z250	Filtek Z250	Thermal cycling (5000 cycles; 5–55°C; dwell time: 20 s)/same	10	G1: DB	SI	SBS	13.85 ± 2.50
					G2: DB	SI+AD (Adper Single Bond 2) (TE)		20.54 ± 4.14
					G3: DB	SI+AD (Single Bond Universal) (SE)		28.29 ± 4.35
					G4: Er; Cr: YSGG laser+PhA	SI		4.75 ± 1.73
					G5: Er; Cr: YSGG laser+PhA	SI+AD (Adper Single Bond 2) (TE)		16.05 ± 2.13
					G6: Er; Cr: YSGG laser+PhA	SI+AD (Single Bond Universal) (TE)		24.61 ± 3.51

Kiomarsi et al. (2017) (b) [32]	Filtek Z250	Filtek Z250	Thermal cycling (10000 cycles; 5–55°C; dwell time: 20 s)/same (5000 cycles)	10	G1: DB+PhA	SI+AD (Adper Single Bond 2) (TE)	SBS	19.08 ± 3.51
					G2: Er, Cr: YSGG laser+PhA	SI+AD (Adper Single Bond 2) (TE)		14.08 ± 2.90

Eliasson and Dahl (2017) [45]	Filtek Supreme XLT	Filtek Supreme XLT	Thermal cycling (5000 cycles; 5–55°C; dwell time: 20 s; transfer time: 3 s)+2 weeks in water/same thermal cycling+6 months in water	66	G1: no treatment	No treatment	*μ*TBS	(Cohesive): 62.2 ± 5.29
				51	G2: AP+PhA	AD (Adper Scotchbond MP) (TE)		27.8 ± 4.04
				65	G3: AP+PhA	AD (Scotchbond Universal) (TE)		33.1 ± 8.55
				60	G4: AP+PhA	AD (Clearfil SE Bond) (SE)		33.4 ± 6.00
				66	G5: AP+PhA	AD (One Step Plus) (TE)		33.9 ± 7.25
				63	G6: AP+PhA	SI+AD (Adper Scotchbond MP) (TE)		33.0 ± 5.65
				65	G7: AP+PhA	SI+AD (Scotchbond Universal) (TE)		51.3 ± 9.57
				61	G8: AP+PhA	SI+AD (Clearfil SE) (SE)		49.4 ± 8.30
				62	G9: AP+PhA	SI+AD (One Step Plus) (TE)		49.5 ± 8.17

Wiegand et al. (2015) [29]	Filtek Supreme XTE	Filtek Supreme XTE	Thermal cycling (5000 cycles; 5–55°C; dwell time: 20 s; transfer time: 10 s)/same	12	G1: AP	AD (OptiBond FL) (TE)	SBS	8.8 ± 2.7
					G2: AP+DB	AD (OptiBond FL) (TE)		23.8 ± 5.5
					G3: AP+AA	AD (OptiBond FL) (TE)		19.9 ± 4.6
					G4: AP+SC	SI+AD (OptiBond FL) (TE)		21.8 ± 6.5

Musa and Nahedh (2014) [52]	Aelite LS Posterior	Filtek Z250	4 weeks in water/thermal cycling (5000 cycles; 5–55°C; dwell time: 5 s)	12	G1: no treatment	No treatment	SBS	(Aelite): 13.77 ± 4.14
					G2: no treatment	AD (One Step Plus) (TE)		(Aelite): 15.80 ± 4.63
					G3: no treatment	AD (Adper Single Bond 2) (TE)		(Filtek Z250): 19.61 ± 1.22
	Filtek Z250	Filtek Z250		12	G4: no treatment	AD (Adper Single Bond 2) (TE)		(Filtek Z250): 17.79 ± 2.64

Eliasson et al. (2014) [30]	Tetric Evo Ceram	Tetric Evo Ceram	Thermal cycling (5000 cycles; 5–55°C; dwell time: 20 s; transfer time: 3 s)/same thermocycling +12 months in water	44	G1: PhA	No treatment	*μ*TBS	(Cohesive): 49.6 ± 5.1
				52	G2: PhA+AP	AD (AdheSE One) (SE)		24.1 ± 7.3
				40	G3: PhA+AP	AD (Clearfil SE) (SE)		33.6 ± 8.4
				40	G4: PhA+AP	AD (Adper Scotchbond MP) (TE)		21.2 ± 9.9
				41	G5: PhA+AP+SC	AD (AdheSE One) (SE)		32.9 ± 8.5
				61	G6: PhA+AP+SC	AD (Clearfil SE) (SE)		36.8 ± 10.7
				58	G7: PhA+AP+SC	AD (Adper Scotchbond MP) (TE)		30.4 ± 8.3
				57	G8: PhA+AP	SI+AD (AdheSE One) (SE)		33.8 ± 6.6
				59	G9: PhA+AP	SI+AD (Clearfil SE) (SE)		41.3 ± 7.5
				53	G10: PhA+AP	SI+AD (Adper Scotchbond MP) (TE)		28.2 ± 6.2

El-Askary et al. (2012) [47]	Grandio Caps	Grandio Caps	1 month in water/same	10	G1: DB+PhA	AD (Solobond Plus) (TE)	TBS	16.9 ± 5.3
					G2: DB+PhA	SI+AD (Solobond Plus) (TE)		12.3 ± 2.5

Staxrud and Dahl (2011) [21]	Filtek Z 250 Charisma Filtek Supreme XT CeramX Mono Tetric Evo Ceram	Filtek Z 250 Charisma Filtek Supreme XT CeramX Mono Tetric Evo Ceram	60 days in water (37°C)/thermal cycling (5000 cycles; 5–55°C; dwell time: 20 s; transfer time: 2-3 s)	10	G1: AP	AD (Adper Scotchbond MP) (TE)	SBS	Filtek Z 250: 22.7 ± 4.3
					G2: AP	No treatment		Charisma: 16.8 ± 4.7
					G3: AP	AD (Scotchbond 1XT) (TE)		Filtek supreme XT: 19.0 ± 5.6
					G4: AP	AD (Xeno III) (SE)		CeramX mono: 16.6 ± 4.4
					G5: AP	AD (AdheSE) (TE)		Tetric Evo Ceram: 16.0 ± 9.8

Papacchini et al. (2007) [44]	Gradia Direct Anterior	Gradia Direct Anterior	1 month in 0.9% saline (37°C)/thermal cycling (5000 cycles; 5–55°C; dwell time: 30 s; transfer time: 5 s)	5	G1: AA+PhA	AD (Adper Scotchbond MP) (TE)	*μ*TBS	32.8 ± 6.6
					G2: AA+PhA	FRC (Filtek Supreme XT Flow)		48.2 ± 7.5
					G3: AA+PhA	FRC (Gradia LoFlo)		43.9 ± 7.7
					G4: AA+PhA	SI		28.3 ± 9.6
					G5: AA+PhA	SI+AD (Clearfil New) (TE)		34.7 ± 11.2
					G6: AA+PhA	SI+AD (Clearfil SE) (SE)		38.0 ± 9.5
					G7: AA+PhA	SI+AD (Clearfil Tri-S) (SE)		33.7 ± 13.0

MPa: mega Pascal; AA: air abrasion with Al_2_O_3_; PhA: (35%/37% Phosphoric Acid); DB: Diamond Bur; AP: Abrasive Paper; SC: Air abrasion with silica coated Al2O3; AR: Argon plasma; AD: AD; SE: Self-Etch; TE: Total-Etch; SI: Silane; FRC: flowable resin composite.

**Table 2:** Surface treatment ratings by corresponding *P* scores.

Rank	(micro)tensile		(micro)shear	
	Treatment	*P* score	Treatment	*P* score
1	Cohesive	0.9627	DB+SI+SE	0.9946
2	AA+FRC	0.9357	DB+SI+TE	0.9552
3	DB+AA+SI+TE	0.8707	LA+SI+TE	0.9288
4	SC+SI+TE	0.8605	DB+SI	0.8797
5	DB+SI+TE	0.7727	LA+SI	0.7505
6	AA+SI+SE	0.7562	SC+SE	0.7414
7	DB+SI+SE	0.7369	AA+SE	0.7021
8	AA+SI+TE	0.7214	DB+SE	0.6986
9	SC+SI+SE	0.7152	AA+SI+TE	0.6364
10	AP+SI+SE	0.6581	SC+TE	0.6362
11	DB+TE	0.6384	SC+SI+TE	0.6210
12	AA+SE	0.6252	SC	0.6078
13	SC+SE	0.6105	AA+AR+SI+TE	0.5644
14	AA+TE	0.5486	AA+TE	0.5633
15	SC+TE	0.4289	DB+TE	0.5022
16	AP+SI+TE	0.4288	AA	0.4526
17	DB+SE	0.4257	SE	0.4190
18	AA+SI	0.3671	AA+AR+TE	0.4164
19	AP+SE	0.2988	AR+SI+TE	0.4026
20	SC	0.2964	AR+TE	0.3197
21	AP+TE	0.2207	DB	0.3039
22	AA	0.2113	AP+TE	0.2936
23	SI+TE	0.1402	AP+SE	0.1400
24	TE	0.1289	AP	0.1222
25	DB	0.0538	AR+SI	0.1159
26	AP	0.0437	TE	0.0864
27	No	0.0431	No	0.0808
28	—	—	AR	0.0647

AP: abrasive paper; No: no surface treatment; AA: air abrasion with Al_2_O_3_ particles; SI: silane; TE: total-etch adhesive; SE: self-etch adhesive; AR: argon plasma; DB: diamond bur; SC: air abrasion with silica-coated Al_2_O_3_ particles; FRC: flowable resin composite; LA: laser.

## Data Availability

The data used to support the findings of this study are available from the corresponding author upon request.

## Supplementary Materials

Supplementary 1 PRISMA checklist for reporting network meta-analysis.

Supplementary 2 search strategies in the databases.

Supplementary 3 list of excluded articles with reasons.

Supplementary 4 risk of bias assessment of individual studies.

Supplementary 5 league tables (two-by-two comparisons).

Supplementary 6 node-split inconsistency results.

## References

[1] Beck F., Lettner S., Graf A. (2015). Survival of direct resin restorations in posterior teeth within a 19-year period (1996–2015): a meta-analysis of prospective studies. *Dental Materials*.

[2] Gordan V. V., Shen C., Riley J., Mjör I. A. (2006). Two-year clinical evaluation of repair versus replacement of composite restorations. *Journal of Esthetic and Restorative Dentistry*.

[3] Negreiros W. M., Ayres A. P. A., Willers A. E., Hirata R., Giannini M. (2021). Effect of argon plasma on repair bond strength using nanofilled and microhybrid composites. *Journal of Esthetic and Restorative Dentistry*.

[4] Özcan M., Pekkan G. (2013). Effect of different adhesion strategies on bond strength of resin composite to composite-dentin complex. *Operative Dentistry*.

[5] Casagrande L., Laske M., Bronkhorst E. M., Huysmans M. C. D. N. J. M., Opdam N. J. M. (2017). Repair may increase survival of direct posterior restorations – a practice based study. *Journal of Dentistry*.

[6] Kanzow P., Wiegand A. (2020). Retrospective analysis on the repair vs. replacement of composite restorations. *Dental Materials*.

[7] Opdam N. J. M., Bronkhorst E. M., Loomans B. A. C., Huysmans M.-C. D. N. J. M. (2012). Longevity of repaired restorations: a practice based study. *Journal of Dentistry*.

[8] Estay J., Martín J., Viera V. (2018). 12 years of repair of amalgam and composite resins: a clinical study. *Operative Dentistry*.

[9] Flury S., Dulla F. A., Peutzfeldt A. (2019). Repair bond strength of resin composite to restorative materials after short- and long-term storage. *Dental Materials*.

[10] Brendeke J., Ozcan M. (2007). Effect of physicochemical aging conditions on the composite-composite repair bond strength. *The Journal of Adhesive Dentistry*.

[11] Kanzow P., Hoffmann R., Tschammler C., Kruppa J., Rödig T., Wiegand A. (2017). Attitudes, practice, and experience of German dentists regarding repair restorations. *Clinical Oral Investigations*.

[12] Kanzow P., Dieckmann P., Hausdörfer T., Attin T., Wiegand A., Wegehaupt F. J. (2017). Repair restorations: questionnaire survey among dentists in the Canton of Zurich, Switzerland. *Swiss Dental Journal*.

[13] Curtis A. R., Shortall A. C., Marquis P. M., Palin W. M. (2008). Water uptake and strength characteristics of a nanofilled resin-based composite. *Journal of Dentistry*.

[14] Ferracane J. L. (2006). Hygroscopic and hydrolytic effects in dental polymer networks. *Dental Materials*.

[15] Vankerckhoven H., Lambrechts P., van Beylen M., Davidson C. L., Vanherle G. (1982). Unreacted methacrylate groups on the surfaces of composite resins. *Journal of Dental Research*.

[16] Ruyter I. E. (1981). Unpolymerized surface layers on sealants. *Acta Odontologica Scandinavica*.

[17] Demarco F. F., Corrêa M. B., Cenci M. S., Moraes R. R., Opdam N. J. (2012). Longevity of posterior composite restorations: not only a matter of materials. *Dental Materials*.

[18] Opdam N. J., Bronkhorst E. M., Loomans B. A., Huysmans M. C. (2010). 12-year survival of composite vs. amalgam restorations. *Journal of Dental Research*.

[19] van de Sande F. H., Opdam N. J., Rodolpho P. A., Correa M. B., Demarco F. F., Cenci M. S. (2013). Patient risk factors' influence on survival of posterior composites. *Journal of Dental Research*.

[20] Peterson J., Rizk M., Hoch M., Wiegand A. (2018). Bonding performance of self-adhesive flowable composites to enamel, dentin and a nano-hybrid composite. *Odontology*.

[21] Staxrud F., Dahl J. E. (2011). Role of bonding agents in the repair of composite resin restorations. *European Journal of Oral Sciences*.

[22] Burrer P., Costermani A., Par M., Attin T., Tauböck T. T. (2021). Effect of varying working distances between sandblasting device and composite substrate surface on the repair bond strength. *Materials*.

[23] Karadas M. (2022). Evaluation of bonding strength of universal adhesives to aged composite resin. *Journal of Adhesion Science and Technology*.

[24] Michelotti G., Niedzwiecki M., Bidjan D. (2020). Silane effect of universal adhesive on the composite-composite repair bond strength after different surface pretreatments. *Polymers (Basel)*.

[25] Dieckmann P., Baur A., Dalvai V., Wiedemeier D. B., Attin T., Tauböck T. T. (2020). Effect of composite age on the repair bond strength after different mechanical surface pretreatments. *The Journal of Adhesive Dentistry*.

[26] Kouros P., Koliniotou-Koumpia E., Spyrou M., Koulaouzidou E. (2018). Influence of material and surface treatment on composite repair shear bond strength. *Journal of Conservative Dentistry*.

[27] Willers A. E., Almeida Ayres A. P., Hirata R., Giannini M. (2022). Effect of universal adhesive application on bond strength of four-year aged composite repair. *Journal of Adhesion Science and Technology*.

[28] Moura D. M. D., Dal Piva A. M. O., Januário A. (2020). Repair bond strength of a CAD/CAM nanoceramic resin and direct composite resin: effect of aging and surface conditioning methods. *The Journal of Adhesive Dentistry*.

[29] Wiegand A., Stucki L., Hoffmann R., Attin T., Stawarczyk B. (2015). Repairability of CAD/CAM high-density PMMA- and composite-based polymers. *Clinical Oral Investigations*.

[30] Eliasson S. T., Tibballs J., Dahl J. E. (2014). Effect of different surface treatments and adhesives on repair bond strength of resin composites after one and 12 months of storage using an improved microtensile test method. *Operative Dentistry*.

[31] Kiomarsi N., Espahbodi M., Chiniforush N., Karazifard M. J., Kamangar S. S. H. (2017). In vitro evaluation of repair bond strength of composite: effect of surface treatments with bur and laser and application of universal adhesive. *Laser Therapy*.

[32] Kiomarsi N., Saburian P., Chiniforush N., Karazifard M. J., Hashemikamangar S. S. (2017). Effect of thermocycling and surface treatment on repair bond strength of composite. *Journal of Clinical and Experimental Dentistry*.

[33] Van Meerbeek B., Peumans M., Poitevin A. (2010). Relationship between bond-strength tests and clinical outcomes. *Dental Materials*.

[34] Valente L. L., Sarkis-Onofre R., Gonçalves A. P., Fernández E., Loomans B., Moraes R. R. (2016). Repair bond strength of dental composites: systematic review and meta-analysis. *International Journal of Adhesion and Adhesives*.

[35] Higgins J. P. T., Thomas J., Chandler J. *Cochrane Handbook for Systematic Reviews of Interventions version 6.0*.

[36] Hutton B., Salanti G., Caldwell D. M. (2015). The PRISMA extension statement for reporting of systematic reviews incorporating network meta-analyses of health care interventions: checklist and explanations. *Annals of Internal Medicine*.

[37] Hadilou M., Dolatabadi A., Ghojazadeh M., Hosseinifard H., Alizadeh Oskuee P., Pournaghi Azar F. (2022). Which surface treatment improves the long-term repair bond strength of aged methacrylate-based composite resin restorations? A systematic review and network meta-analysis.

[38] Sterne J. A. C., Savović J., Page M. J. (2019). RoB 2: a revised tool for assessing risk of bias in randomised trials. *British Medical Journal*.

[39] Mendes L. T., Loomans B. A. C., Opdam N. J. M., Silva C. L. D., Casagrande L., Lenzi T. L. (2020). Silane coupling agents are beneficial for resin composite repair: a systematic review and meta-analysis of in vitro studies. *The Journal of Adhesive Dentistry*.

[40] Mcguinness L. A., Higgins J. P. T. (2021). Risk-of-bias VISualization (robvis): an R package and Shiny web app for visualizing risk-of-bias assessments. *Research Synthesis Methods*.

[41] Rücker G., Schwarzer G. (2015). Ranking treatments in frequentist network meta-analysis works without resampling methods. *BMC Medical Research Methodology*.

[42] Higgins J. P. T., Thompson S. G., Deeks J. J., Altman D. G. (2003). Measuring inconsistency in meta-analyses. *BMJ*.

[43] Oglakci B., Arhun N. (2019). The shear bond strength of repaired high-viscosity bulk-fill resin composites with different adhesive systems and resin composite types. *Journal of Adhesion Science and Technology*.

[44] Papacchini F., Toledano M., Monticelli F. (2007). Hydrolytic stability of composite repair bond. *European Journal of Oral Sciences*.

[45] Eliasson S. T., Dahl J. E. (2017). Effect of curing and silanizing on composite repair bond strength using an improved micro-tensile test method. *Acta Biomater Odontol Scand*.

[46] Ayres A., Hirata R., Fronza B. M., Lopes B. B., Ambrosano G., Giannini M. (2019). Effect of argon plasma surface treatment on bond strength of resin composite repair. *Operative Dentistry*.

[47] El-Askary F. S., El-Banna A. H., van Noort R. (2012). Immediate vs delayed repair bond strength of a nanohybrid resin composite. *The Journal of Adhesive Dentistry*.

[48] Ugurlu M., Al-Haj Husain N., Özcan M. (2022). Repair of bulk-fill and nanohybrid resin composites: effect of surface conditioning, adhesive promoters, and long-term aging. *Materials*.

[49] Souza M. O., Leitune V. C., Rodrigues S. B., Samuel S. M., Collares F. M. (2017). One-year aging effects on microtensile bond strengths of composite and repairs with different surface treatments. *Brazilian Oral Research*.

[50] Demirel G., Gür G. (2019). Micro-shear bond strength of aged resin composite repaired with different universal adhesives. *Meandros Medical and Dental Journal*.

[51] Kanzow P., Baxter S., Rizk M., Wassmann T., Wiegand A. (2019). Effectiveness of a universal adhesive for repair bonding to composite and amalgam. *Journal of Oral Science*.

[52] Al Musa A. H., Al Nahedh H. N. (2014). Incremental layer shear bond strength of low-shrinkage resin composites under different bonding conditions. *Operative Dentistry*.

[53] Chuenweravanich J., Kuphasuk W., Saikaew P., Sattabanasuk V. (2022). Bond durability of a repaired resin composite using a universal adhesive and different surface treatments. *The Journal of Adhesive Dentistry*.

[54] Baldissera R. A., Corrêa M. B., Schuch H. S. (2013). Are there universal restorative composites for anterior and posterior teeth?. *Journal of Dentistry*.

[55] Da Rosa Rodolpho P. A., Donassollo T. A., Cenci M. S. (2011). 22-year clinical evaluation of the performance of two posterior composites with different filler characteristics. *Dental Materials*.

[56] Demarco F. F., Collares K., Coelho-de-Souza F. H. (2015). Anterior composite restorations: a systematic review on long-term survival and reasons for failure. *Dental Materials*.

[57] Morresi A. L., D'Amario M., Capogreco M. (2014). Thermal cycling for restorative materials: does a standardized protocol exist in laboratory testing? A literature review. *Journal of the Mechanical Behavior of Biomedical Materials*.

[58] International Organization for Standardization (2003). Dental materials – testing of adhesion to tooth structure.

[59] Mjör I. A., Shen C., Eliasson S. T., Richter S. (2002). Placement and replacement of restorations in general dental practice in Iceland. *Operative Dentistry*.

[60] Szczesio-Wlodarczyk A., Fronczek M., Ranoszek-Soliwoda K., Grobelny J., Sokolowski J., Bociong K. (2022). The first step in standardizing an artificial aging protocol for dental composites—evaluation of basic protocols. *Molecules*.

[61] Ghavami-Lahiji M., Firouzmanesh M., Bagheri H., Jafarzadeh Kashi T. S., Razazpour F., Behroozibakhsh M. (2018). The effect of thermocycling on the degree of conversion and mechanical properties of a microhybrid dental resin composite. *Restorative Dentistry & Endodontics*.

[62] Inoue S., Koshiro K., Yoshida Y. (2005). Hydrolytic stability of self-etch adhesives bonded to dentin. *Journal of Dental Research*.

[63] Shono Y., Terashita M., Shimada J. (1999). Durability of resin-dentin bonds. *The Journal of Adhesive Dentistry*.

[64] Moura D. M. D., Veríssimo A. H., Leite Vila-Nova T. E., Calderon P. S., Özcan M., Assunção Souza R. O. (2022). Which surface treatment promotes higher bond strength for the repair of resin nanoceramics and polymer-infiltrated ceramics? A systematic review and meta- analysis. *The Journal of Prosthetic Dentistry*.

[65] Staxrud F., Tveit A. B., Rukke H. V., Kopperud S. E. (2016). Repair of defective composite restorations. A questionnaire study among dentists in the Public Dental Service in Norway. *Journal of Dentistry*.

