# Supplementary material for: Effect of Different Surface Treatments on the Long-Term Repair Bond Strength of Aged Methacrylate-Based Resin Composite Restorations: A Systematic Review and Network Meta-analysis
Source: Biomed Res Int. 2022 Sep 5;2022:7708643. doi: 10.1155/2022/7708643 (PMC10643039; doi:10.1155/2022/7708643)
Supplement: Supplementary 2 — search strategies in the databases. [file 7708643.f2.docx]

**Supplementary file 2. Search strategies in the databases.**

**PUBMED**

(((repair*[Text Word]) OR (reparation[Text Word])) AND ((((("Composite Resins"[MeSH Terms]) OR (Composite Resin*[Text Word])) OR (Resin, Composit*[Text Word])) OR ("Bisphenol A-Glycidyl Methacrylate"[Mesh])) OR (methacrylic resin*[Text Word]))) AND ("Dentistry"[Mesh] OR dent*)

**Embase**

('resin'/exp OR 'composite resin*':ti,ab OR 'resin, composit*':ti,ab OR 'bisphenol a-glycidyl methacrylate':ti,ab OR 'methacrylic resin*':ti,ab) AND ('repair*':ti,ab OR 'reparation':ti,ab) AND ('dentistry'/exp OR 'dentistry' OR 'dent*' OR 'dent*':ti,ab)

**Scopus**

( TITLE-ABS-KEY ( "resin*" OR "Composite Resin*" OR "Resin Composit*" OR {Bisphenol A Glycidyl Methacrylate} OR "methacrylic resin*" ) ) AND ( TITLE-ABS-KEY ( "repair*" OR "reparation" ) ) AND ( LIMIT-TO ( SUBJAREA , "DENT" ) )

**Web of Science**

---------------------------------------------------------------#1--------------------------------------------------------------------------
TS=("resin*" OR "Composite Resin*" OR "Resin Composit*" OR "Bisphenol A Glycidyl Methacrylate" OR "methacrylic resin*")

---------------------------------------------------------------#2--------------------------------------------------------------------------

TS=("repair*" OR "reparation")

---------------------------------------------------------------#3--------------------------------------------------------------------------

TS=('dent*')

---------------------------------------------------------------#3--------------------------------------------------------------------------

#3 AND #2 AND #1
