# Supplementary figures and images for: Effect of Different Surface Treatments on the Long-Term Repair Bond Strength of Aged Methacrylate-Based Resin Composite Restorations: A Systematic Review and Network Meta-analysis
Source: Biomed Res Int. 2022 Sep 5;2022:7708643. doi: 10.1155/2022/7708643 (PMC10643039; doi:10.1155/2022/7708643)

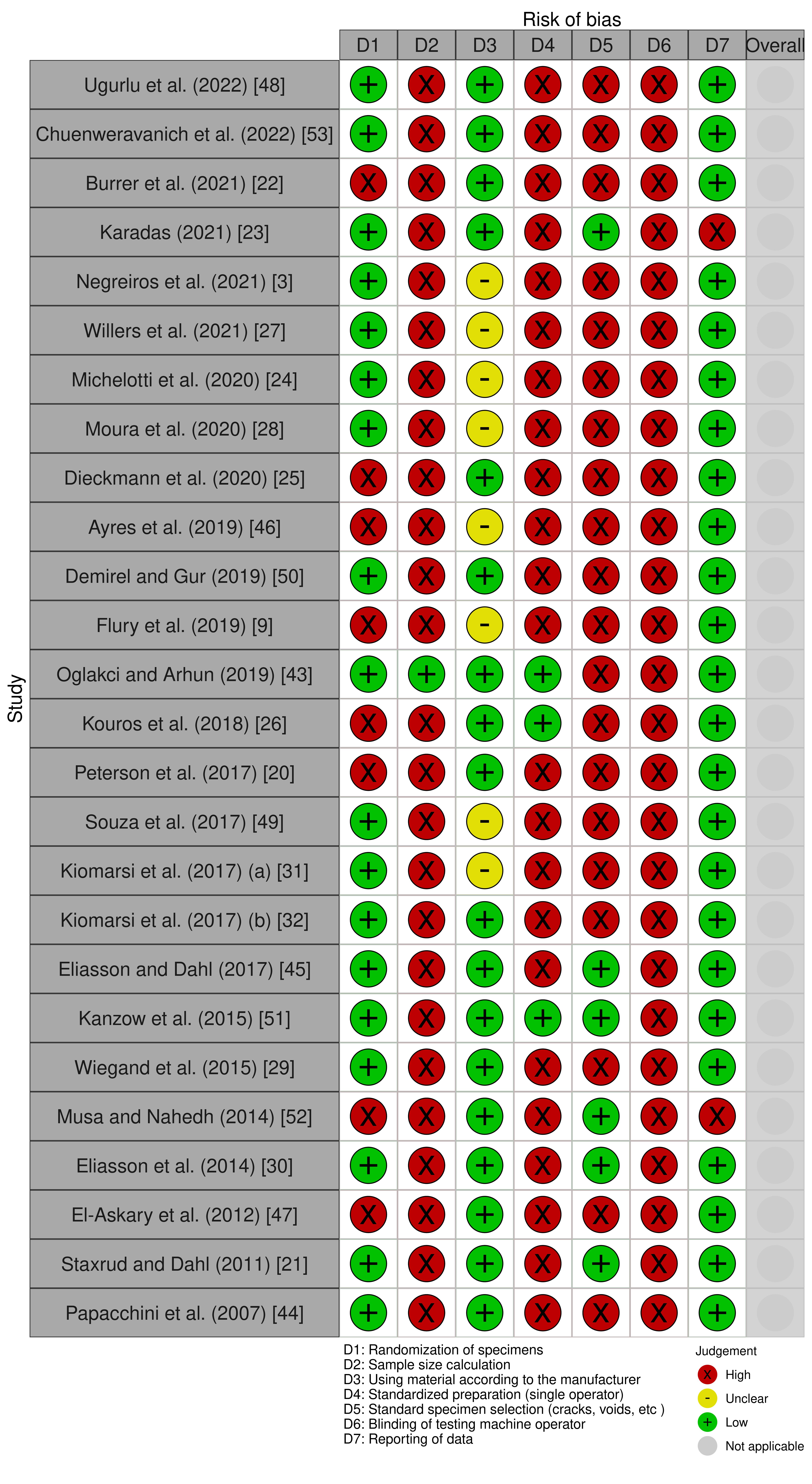

Supplement: Supplementary 4 — risk of bias assessment of individual studies. [file 7708643.f4.docx]

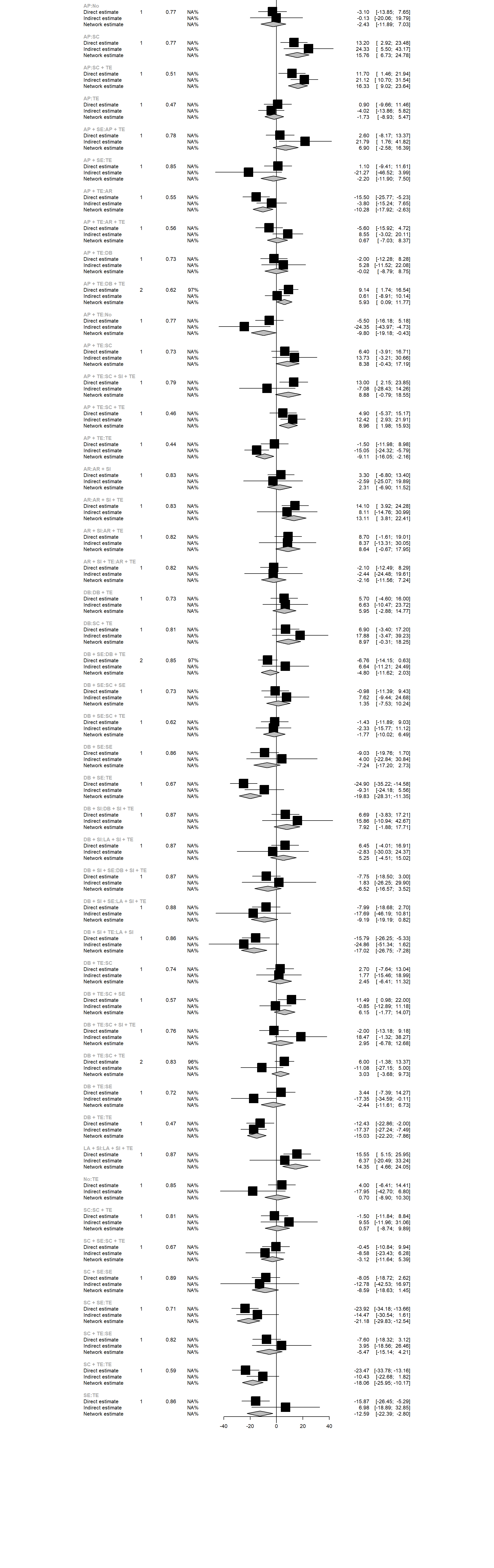

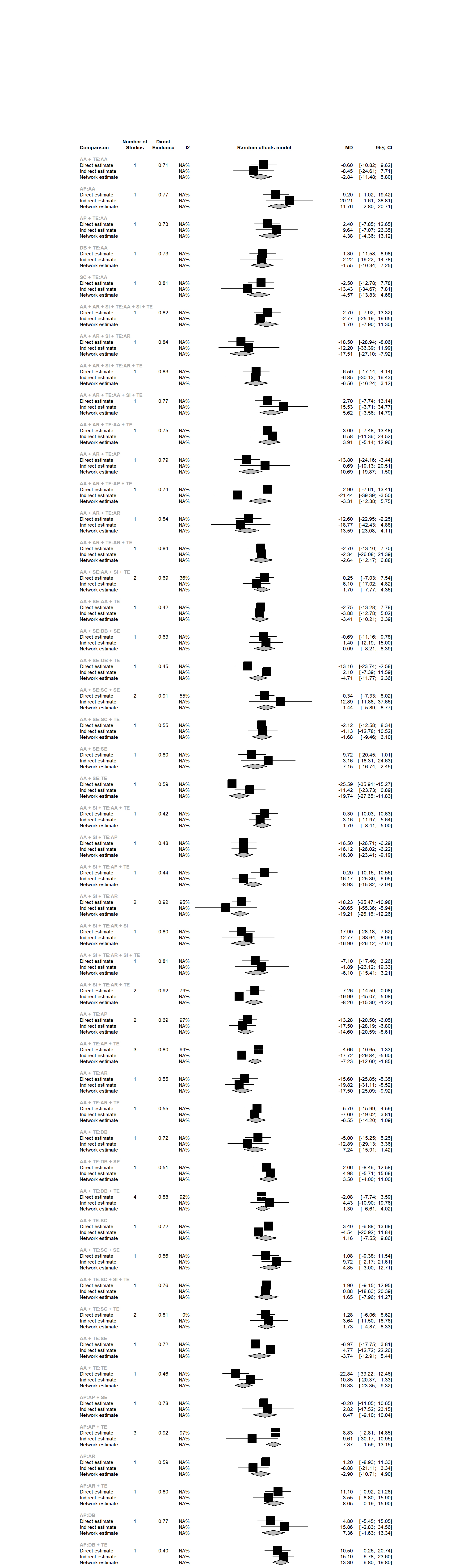
Cccc

(micro)Shear


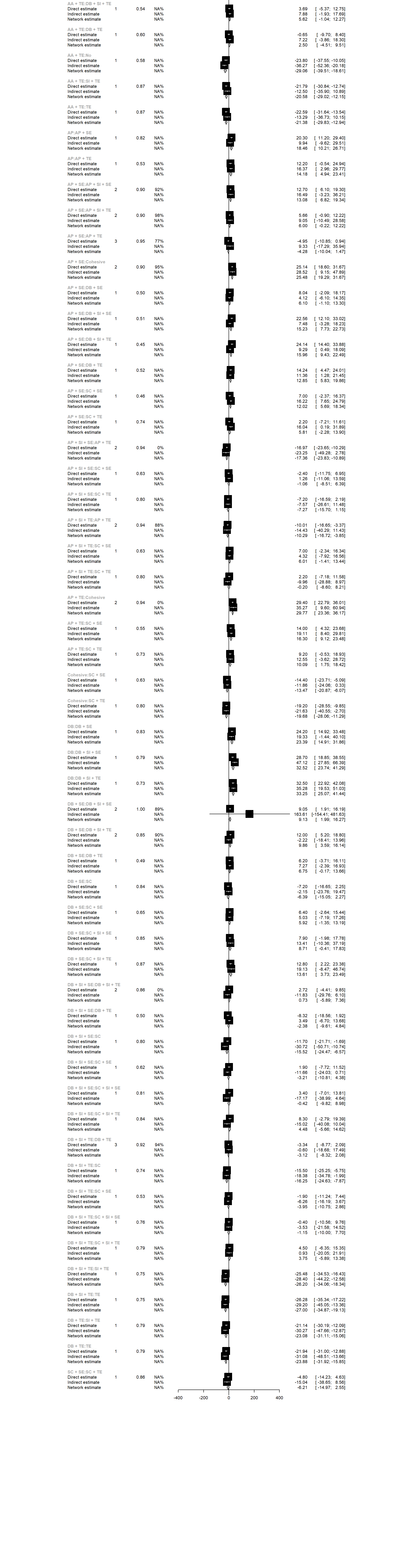

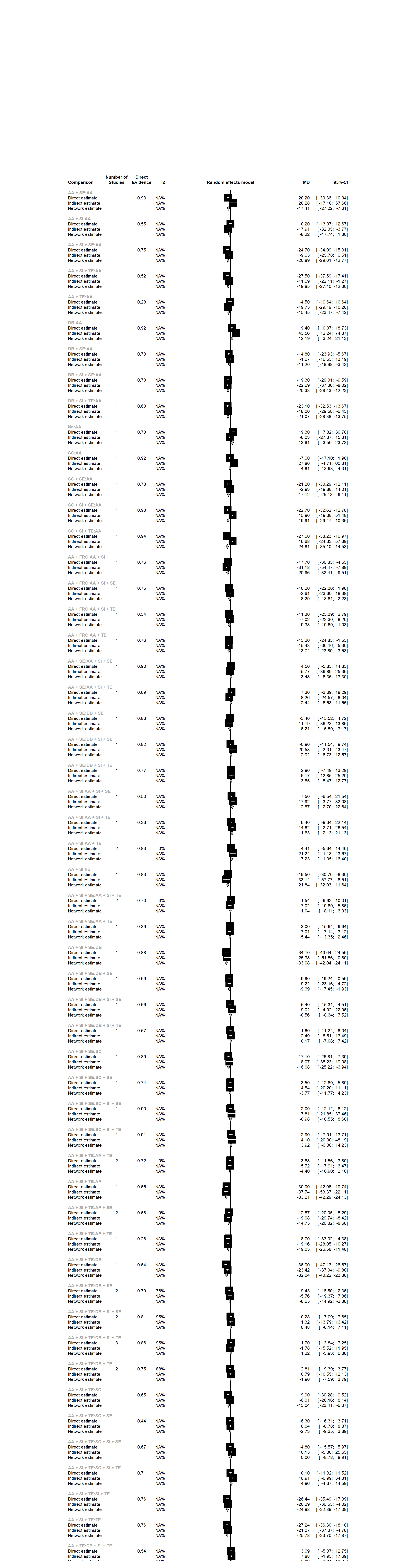


(micro)Tensile


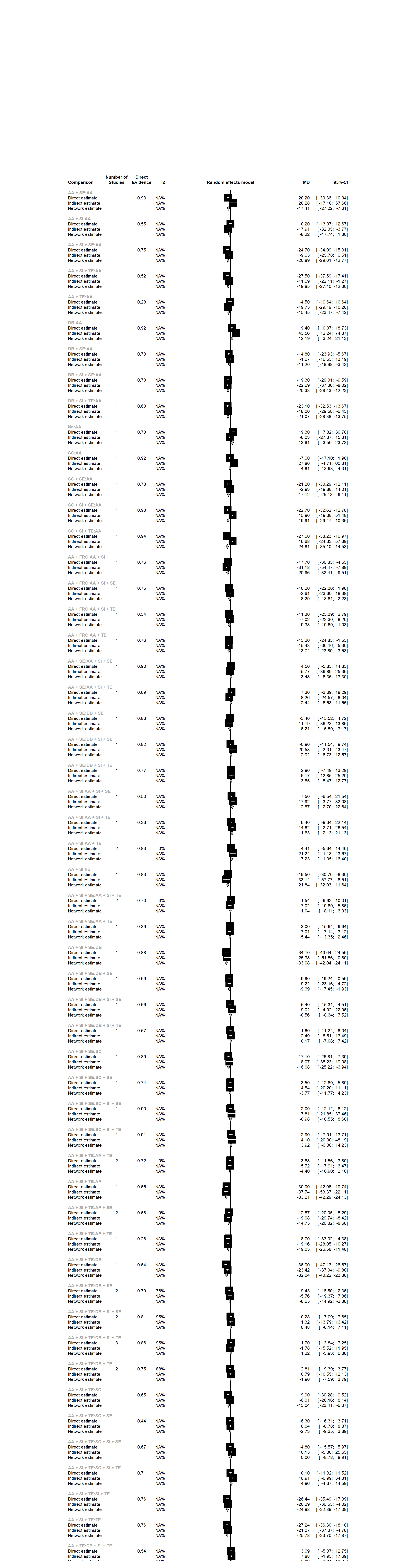

Supplement: Supplementary 6 — node-split inconsistency results. [file 7708643.f6.docx]
